# Schisandrin C Affects Glucose-Stimulated Insulin Secretion in Pancreatic β-Cells and Glucose Uptake in Skeletal Muscle Cells

**DOI:** 10.3390/molecules26216509

**Published:** 2021-10-28

**Authors:** Dahae Lee, Young-Mi Kim, Hyun Woo Kim, You-Kyoung Choi, Bang Ju Park, Sang Hoon Joo, Ki Sung Kang

**Affiliations:** 1 College of Korean Medicine, Gachon University, Seongnam 13120, Korea; pjsldh@gachon.ac.kr; 2 Research Institute of Pharmaceutical Sciences and College of Pharmacy, Seoul National University, Gwanak-ro, Gwanak-gu, Seoul 08826, Korea; 0210121@hanmail.net (Y.-M.K.); hwkim8906@gmail.com (H.W.K.); 3 Department of Korean International Medicine, College of Korean Medicine, Gachon University, Seongnam 13120, Korea; kosmos@gachon.ac.kr; 4 Department of Electronic Engineering, Gachon University, Seongnam 13120, Korea; sooyong1320@gachon.ac.kr; 5 College of Pharmacy, Daegu Catholic University, Gyeongsan 38430, Korea

**Keywords:** schisandrin C, glucose-stimulated insulin secretion, glucose uptake, PDX-1, GLUT-4

## Abstract

The aim of our study was to investigate the effect of three lignans (schisandrol A, schisandrol B, and schisandrin C) on insulin secretion in rat INS-1 pancreatic β-cells and glucose uptake in mouse C2C12 skeletal muscle cells. Schisandrol A and schisandrin C enhanced insulin secretion in response to high glucose levels with no toxic effects on INS-1 cells. The effect of schisandrin C was superior to that of gliclazide (positive control), a drug commonly used to treat type 2 diabetes (T2D). In addition, western blot analysis showed that the expression of associated proteins, including peroxisome proliferator-activated receptor γ (PPARγ), pancreatic and duodenal homeobox 1 (PDX-1), phosphatidylinositol 3-kinase (PI3K), Akt, and insulin receptor substrate-2 (IRS-2), was increased in INS-1 cells after treatment with schisandrin C. In addition, insulin secretion effect of schisandrin C were enhanced by the Bay K 8644 (L-type Ca^2+^ channel agonist) and glibenclamide (K^+^ channel blocker), were abolished by the nifedipine (L-type Ca^2+^ channel blocker) and diazoxide (K^+^ channel activator). Moreover, schisandrin C enhanced glucose uptake with no toxic effects on C2C12 cells. Western blot analysis showed that the expression of associated proteins, including insulin receptor substrate-1 (IRS-1), AMP-activated protein kinase (AMPK), PI3K, Akt, glucose transporter type 4 (GLUT-4), was increased in C2C12 cells after treatment with schisandrin C. Schisandrin C may improve hyperglycemia by enhancing insulin secretion in pancreatic β-cells and improving glucose uptake into skeletal muscle cells. Our findings may provide evidence that schisandrin C may be beneficial in devising novel anti-T2D strategies.

## 1. Introduction

Type 2 diabetes (T2D) is a common metabolic disorder with a steadily increasing global prevalence. It is principally characterized by decreased insulin secretion [1], which involves the mechanism mainly known as glucose-stimulated insulin secretion (GSIS). GSIS plays a key role in regulating normal homeostatic levels [2]. In the development of T2D, impaired GSIS is associated with β-cell dysfunction, whereas robust GSIS after meals prevents hyperglycemia. In particular, impaired GSIS in obesity and insulin-resistant conditions may underlie the transition to T2D [3]. In addition, in the T2D, skeletal muscle as a main site for insulin-induced glucose uptake after glucose intake become resistance to the action of insulin, which leads to impaired glucose uptake [4]. Thus, researching anti-T2D drugs based on understanding the mechanisms involved in GSIS in pancreatic β-cells and glucose uptake in skeletal muscle cells could be the foundation of prevention strategies for T2D.

Lignans have gained increasing attention as accumulating evidence suggests their effects against diabetes. Lignans are found in many plants, especially in the cell wall, but they also exist as minor constituents of other plant parts [5]. One study reported that secoisolariciresinol diglucoside improved glycemic control and insulin sensitivity in T2D patients [6]. Another study reported that the daily administration of five lignans (secoisolariciresinol diglucoside, lariciresinol, matairesinol, pinoresinol, and secoisolariciresinol) improved glucose levels in streptozotocin-induced diabetic rats [7]. In diet-induced obese mice, secoisolariciresinol diglucoside improved pancreatic β-cell function [8]. Four lignans (arctigenin, arctiin, matairesinol, and matairesinoside) protected against alloxan-induced damage in the pancreatic β-cells of zebrafish [9]. A lignan-rich fraction containing schizandrin, gomisin A, and angeloylgomisin H increased insulin secretion in diabetic rats [10]. Also, schisandrin C isolated from *Schisandra chinensis* was found to increase glucose uptake in HepG2 cells [11]. Of the lignans from the fruits of *S. chinensis*, three major lignans (schisandrol A, schisandrol B, and schisandrin C) were selected for this study. Schisandrol A have been reported to possess estrogenic activity (PMID: 34371773), hepatoprotective effect (PMID: 25753323), 5-lipoxygenase inhibition (PMID: 19277963), muscle relaxation effect (PMID: 27064883), neuroprotective effect (PMID: 31629801), and P-gp inhibition (PMID: 17318783). Former pharmacological investigations on schisandrol B have documented various activities of shisandrol B including liver enlargement (PMID: 33662920), amelioration of metastatic melanoma (PMID: 32028184), anti-inflammatory activities (PMID: 29423034, PMID: 24749675, PMID: 24211520), hepatoprotective activity (PMID: 28128437, PMID: 25753323), PCSK9 mRNA expression inhibition (PMID: 28139296), and promotion of liver regeneration (PMID: 29066412). Previous studies on the bioactivity of schisandrin C have indicated that schisandrin C exhibits anti-inflammatory [12,13], anti-cancer activity [14], anti-oxidative activity [15], hepatoprotective activity [PMID: 25753323], inhibitory activity against PCSK9 mRNA expression [PMID: 28139296], lipid-lowering activity [16], and neuroprotective activity (PMID: 26074330, PMID: 20740476) activity. In addition, schisandrin C regulates inflammatory factors in diabetic mice, thereby attenuating nephropathy, which is a major complication of diabetes [17]. Therefore, if its anti-diabetic effect is also proven, its potential as an anti-diabetic drug will increase. However, very few studies on GSIS and its cellular mechanism have been used to evaluate the anti-diabetic effects of lignans. Therefore, we evaluated the insulin enhancement effect of three lignans (schisandrol A, schisandrol B, and schisandrin C) and their mechanisms of action in a rodent insulin-secreting β-cell line. In addition, among the three lignans, the most effective lignan on GSIS was studied for its effect on glucose uptake in skeletal muscle cells.

## 2. Results

### 2.1. Effect of Three Lignans on Glucose-Stimulated Insulin Secretion

**Figure 1:**
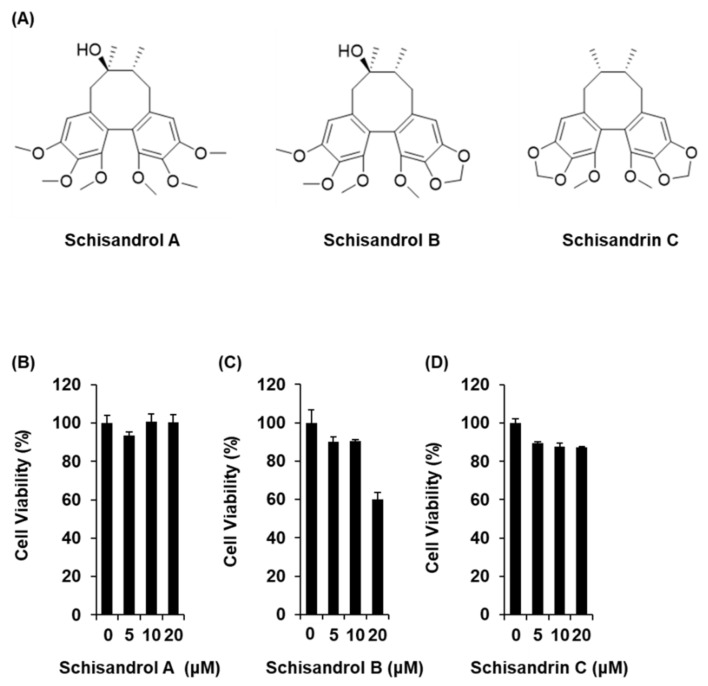
Effects of three lignans (schisandrol A, schisandrol B, and schisandrin C) on the viability of INS-1 cells. (**A**) Chemical structures of the compounds. (**B**–**D**) MTT assay results of the cell viability of INS-1 cells after 24 h treatment with schisandrol A, schisandrol B, and schisandrin C, compared with the control (0 μM).

**Figure 2:**
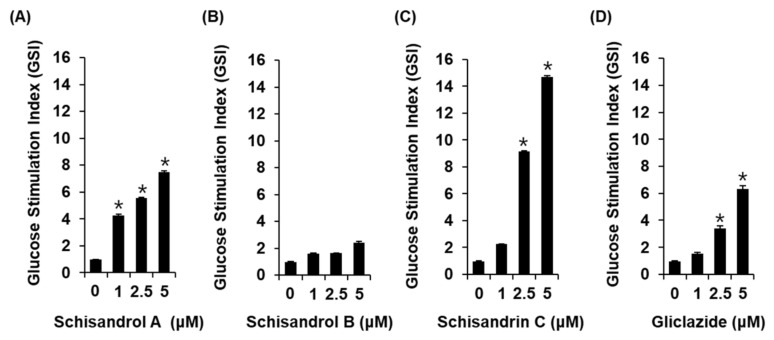
Effects of three lignans (schisandrol A, schisandrol B, and schisandrin C) on glucose-stimulated insulin secretion in INS-1 cells. Insulin secretion in INS-1 cells after 1 h incubation with basal (2.8 mM) and stimulant (16.7 mM) concentrations of glucose in the presence or absence of (**A**–**C**) schisandrol A, schisandrol B, schisandrin C, and (**D**) gliclazide (positive control) assessed by insulin secretion assay. The data represent the mean ± S.E.M., n = 3, * *p* < 0.05 compared with the control.

The three lignans used in the present study (schisandrol A, schisandrol B, and schisandrin C) were obtained from previous studies, and the ^1^H and ^13^C NMR spectroscopic data, as well as the purity results, are provided in the Supplementary Materials [18]. We investigated whether the three lignans could enhance GSIS without cytotoxicity to INS-1 cells. Except for 20 μM schisandrol B, all concentrations of schisandrol A, schisandrol B, and schisandrin C were found to be nontoxic to INS-1 cells (Figure 1B–D). We then confirmed whether schisandrol A, schisandrol B, and schisandrin C at non-toxic concentrations led to an increase in GSIS. As shown in Figure 2A,C, schisandrol A and schisandrin C increased GSIS, expressed as the glucose-stimulated index (GSI). The fold change was set at 1 for the untreated cells. The resultant GSI values were found to be 4.26 ± 0.09, 5.56 ± 0.01, and 7.47 ± 0.11 for schisandrol A at 1 μM, 2.5 μM, and 5 μM, respectively (Figure 2A). GSI values higher than those of schisandrol A were 9.16 ± 0.05 and 14.68 ± 0.11 for schisandrin C at 2.5 μM and 5 μM, respectively (Figure 2C). In addition, the values were superior to those of gliclazide (positive control), which had GSI values of 3.41 ± 0.18 and 6.32 ± 0.25 at 2.5 μM and 5 μM, respectively (Figure 2D). These results suggest that 2.5 μM and 5 μM schisandrin C enhanced insulin secretion in response to high glucose (16.7 mM) while exhibiting no toxic effects on INS-1 cells.

### 2.2. Effect of Schisandrin C on the Protein Expression of PPARγ, P-IRS-2, IRS-2 (Ser731), P-PI3K, PI3K, p-Akt (Ser473), Akt, and PDX-1

**Figure 3:**
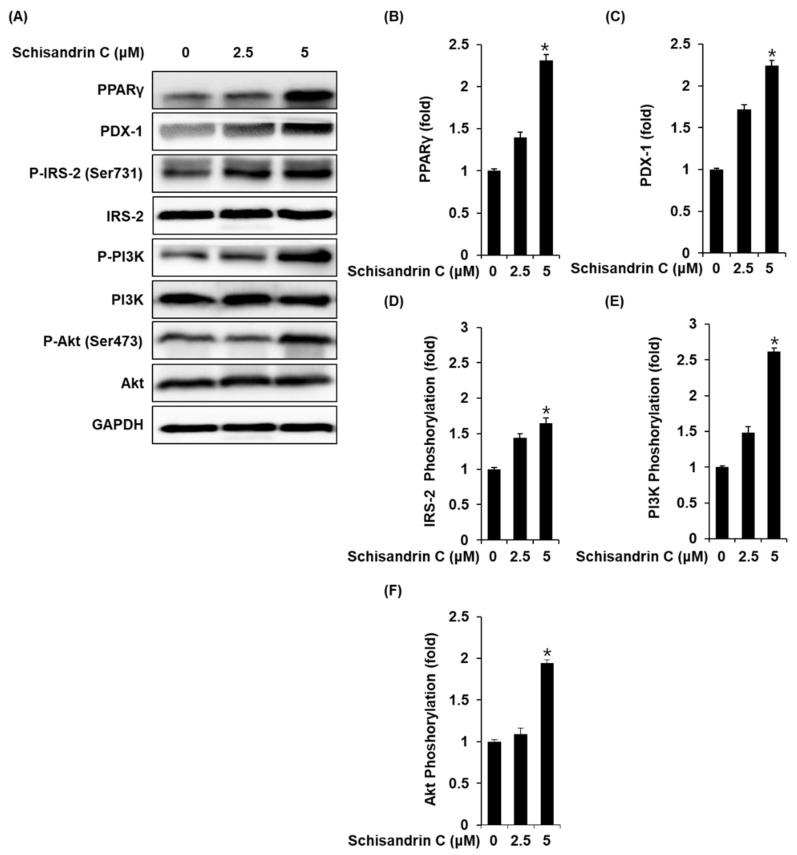
Effect of schisandrin C on the protein expression levels of peroxisome proliferator-activated receptor γ (PPARγ), pancreatic and duodenal homeobox 1 (PDX-1), phospho-insulin receptor substrate-2 (P-IRS-2) (Ser731), IRS-2, phospho-phosphatidylinositol 3-kinase (P-PI3K), PI3K, phospho-Akt (P-Akt) (Ser473), and Akt. (**A**) Protein expression levels of PPARγ, PDX-1, P-IRS-2 (Ser731), IRS-2, P-PI3K, PI3K, P-Akt (Ser473), Akt, and glyceraldehyde 3-phosphate dehydrogenase (GAPDH) in INS-1 cells treated or untreated with 2.5 and 5 μM schisandrin C for 24 h. (**B**–**F**) Each bar graph presents the densitometric quantification of western blot bands. The data represent the mean ± S.E.M., n = 3, * *p* < 0.05 compared with the control.

INS-1 cells treated with 2.5 and 5 μM schisandrin C showed increased protein expression of PPARγ, PDX-1 and phosphorylation levels of IRS-2, PI3K, Akt compared with the untreated controls (Figure 3).

### 2.3. Effect of Schisandrin C on ATP/ADP Ratio and Involvement of L-Type Ca^2+^ and K^+^ Channels

**Figure 4:**
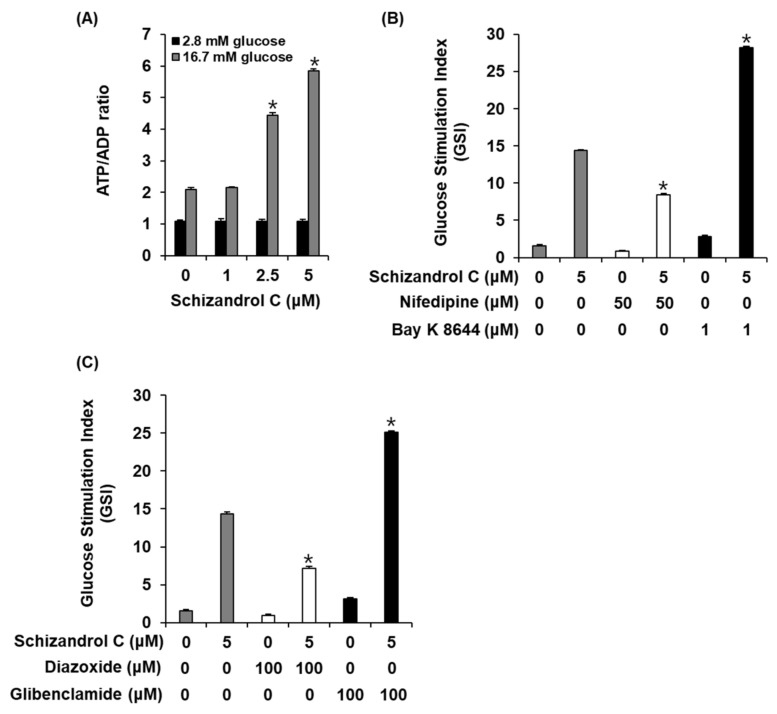
Effect of schisandrin C on ATP/ADP ratio and involvement of L-type Ca^2+^ and K^+^ channels in INS-1 cells. (**A**) ATP/ADP ratio in INS-1 cells after 1 h incubation with basal (2.8 mM) and stimulant (16.7 mM) concentrations of glucose in the presence or absence of schisandrin C assessed by ADP/ATP ratio assay. (**B**) Insulin secretion in INS-1 cells after 1 h incubation with basal (2.8 mM) and stimulant (16.7 mM) concentrations of glucose in the presence or absence of schisandrin C, nifedipine (L-type Ca^2+^ channel blocker), and Bay K 8644 (L-type Ca^2+^ channel activator) assessed by insulin secretion assay. (**C**) Insulin secretion in INS-1 cells after 1 h incubation with basal (2.8 mM) and stimulant (16.7 mM) concentrations of glucose in the presence or absence of schisandrin C, diazoxide (K^+^ channel activator), and glibenclamide (K^+^ channel blocker) assessed by insulin secretion assay. The data represent the mean ± S.E.M., n = 3, * *p* < 0.05 compared with the control.

To evaluate a possibility that schisandrin C enhanced GSIS in INS-1 cells through altering the intracellular ATP/ADP ratio, we measured the effect of schisandrin C on the ATP/ADP ratio. As shown in Figure 4A, schisandrin C increased the glucose-dependent ATP/ADP ratio. In addition, we evaluated the ability of schisandrin C to modulate the K^+^ and Ca^2+^ channels. As shown in Figure 4B,C, insulin secretion effect of schisandrin C were enhanced by the Bay K 8644 (L-type Ca^2+^ channel agonist) and glibenclamide (K^+^ channel blocker), were abolished by the nifedipine (L-type Ca^2+^ channel blocker) and diazoxide (K^+^ channel activator).

### 2.4. Effect of Schisandrin C on Glucose Uptake in Skeletal Muscle Cells

**Figure 5:**
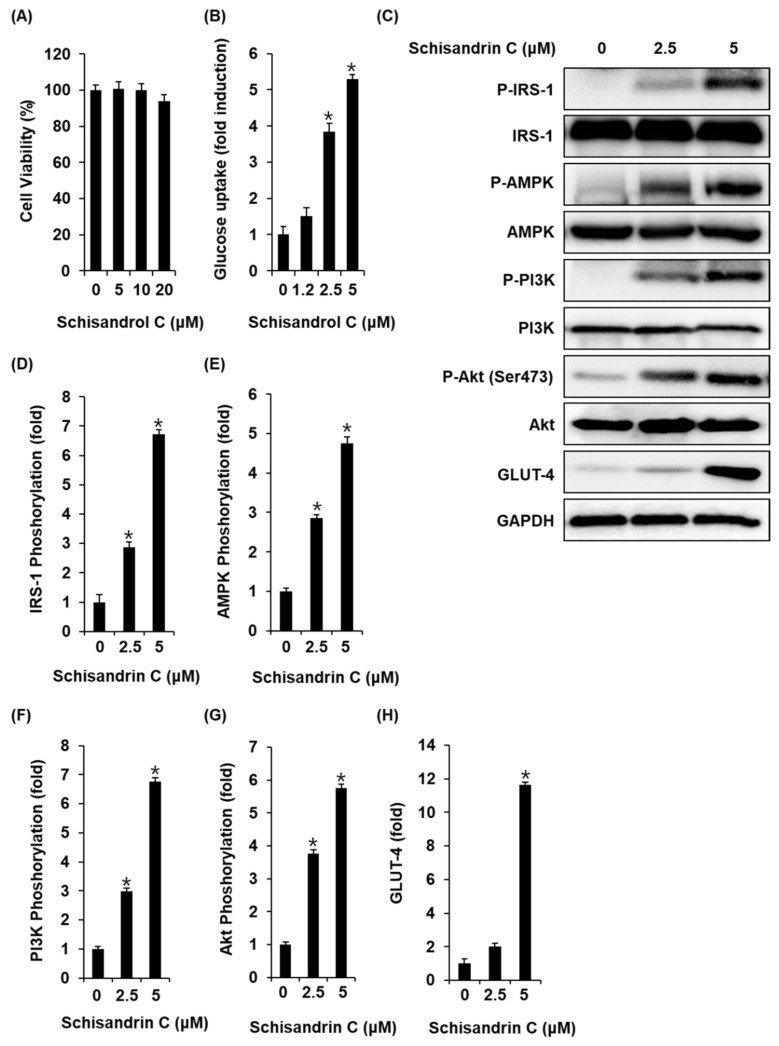
Effects of schisandrin C on the glucose uptake in C2C12 cells. (**A**) A MTT assay result of the cell viability of C2C12 cells after 24 h treatment with schisandrin C compared with the control (0 μM). (**B**) Glucose uptake in C2C12 cells after 1 h incubation with schisandrin C and 2-(N-(7-Nitrobenz-2-oxa-1,3-diazol-4-yl) amino)-2-Deoxyglucose (2-NBDG) assessed by glucose uptake assay. (**C**) Effect of schisandrin C on the protein expression levels of phospho-insulin receptor substrate-1 (P-IRS-1), IRS-1, phospho-AMP-activated protein kinase (P-AMPK), AMPK, phospho-phosphatidylinositol 3-kinase (P-PI3K), PI3K, phospho-Akt (P-Akt) (Ser473), Akt, glucose transporter type 4 (GLUT-4), and glyceraldehyde 3-phosphate dehydrogenase (GAPDH) in INS-1 cells treated or untreated with 2.5 and 5 μM schisandrin C for 24 h. (**D**–**H**) Each bar graph presents the densitometric quantification of western blot bands. The data represent the mean ± S.E.M., n = 3, * *p* < 0.05 compared with the control.

**Figure 6:**
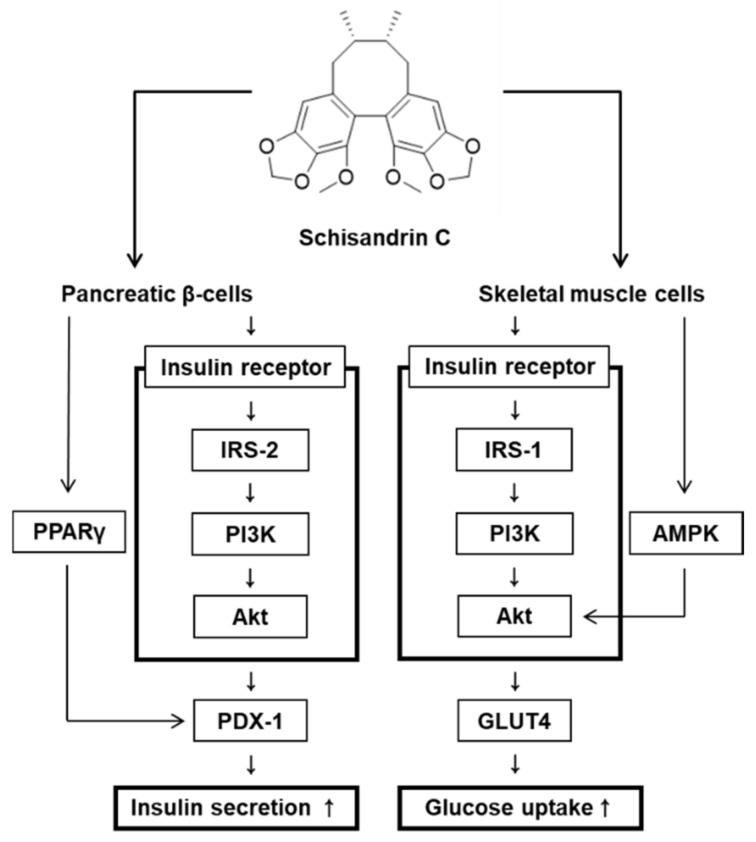
Schematic illustration of the effects of schisandrin C on glucose-stimulated insulin secretion in pancreatic β-cells and glucose uptake in skeletal muscle cells.

We also investigated whether the schisandrin C could enhance glucose uptake without cytotoxicity to C2C12 cells. All concentrations of schisandrin C were found to be nontoxic to C2C12 cells (Figure 5A). We then confirmed whether schisandrin C at non-toxic concentrations led to an increase in glucose uptake activity. As shown in Figure 2B, schisandrin C increased glucose uptake activity presented as fold induction. In addition, C2C12 cells treated with 2.5 μM and 5 μM schisandrin C showed increased protein expression of GLUT-4 and phosphorylation levels of IRS-1, AMPK, PI3K, Akt compared with the untreated controls (Figure 5C–H). A schematic illustration of the proposed mechanisms of the effects of schisandrin C on GSIS in pancreatic β-cells and glucose uptake in skeletal muscle cells was shown in Figure 6.

## 3. Discussion

In the present study, we used cell-based assays to investigate the effect of three lignans (schisandrol A, schisandrol B, and schisandrin C) on GSIS. The concentrations of the three lignans that exhibited no toxicity were used in the GSIS assay. Schisandrol A and schisandrin C increased the GSIS. Schisandrin C, which exhibited the greatest effect, was superior to gliclazide, a drug commonly used to treat T2D in the GSI values. Schisandrol B had no effect on GSIS. It was associated with the ability of schisandrin C to increase the ATP/ADP ratio. It has been documented that glucose metabolism in pancreatic β-cells requires the increase in the intracellular ATP/ADP ratio that promotes insulin secretion vis Ca^2+^ influx, closure of ATP-sensitive potassium (K_ATP_) channels, and plasma membrane depolarization [19]. Previous studies have reported that Bay K 8644 (a L-type Ca^2+^ channel agonist) and glibenclamide (a K^+^ channel blocker) enhanced insulin secretion in pancreatic β-cells, while the channel opener nifedipine (L-type Ca^2+^ channel blocker) and diazoxide (K^+^ channel activator) inhibited insulin secretion [19,20,21,22]. In our study, insulin secretion effect of schisandrin C were enhanced by the Bay K 8644 and glibenclamide were abolished by nifedipine and diazoxide. These results suggested that schisandrin C impacts other pathways that are dependent on K_ATP_ channels sensitive to glibenclamide and diazoxide and Ca^2+^ channels sensitive to Bay K 8644 and nifedipine.

Previous studies have reported that PPARγ agonists enhance GSIS in INS-1 cells, rat islets, and mouse islets [23,24,25]. These studies indicate that GSIS is usually accompanied by PPARγ activity. In addition, the importance of IRS-2 and PDX-1 has been demonstrated in studies on the insulin secretion effects of phytochemical compounds [26]. For example, IRS-2 knockdown mice displayed impaired GSIS [23]. In addition, phosphorylated IRS-2 can stimulate the PI3K/Akt pathway, which plays an essential role in regulating pancreatic β-cell proliferation and mass expansion [27]. Reduced Akt activity leads to dysfunction in insulin secretion and insulin synthesis [28]. In contrast, increased Akt activity in rat islets upregulates the nuclear translocation of PDX-1, a transcription promoter of preproinsulin [29]. Accumulating evidence indicates that PDX-1 knockdown mice display impaired GSIS and islet function [30,31]. Therefore, enhanced expression of PPARγ, PDX-1, IRS-2, PI3K, and Akt are closely related to the normal function of pancreatic β-cells and GSIS, which is consistent with the present results. In the present study, INS-1 cells treated with schisandrin C showed increased protein expression of PPARγ, PDX-1, IRS-2, PI3K, and Akt compared with the untreated controls. Therefore, when the expression of these proteins is enhanced after treatment with schisandrin C, GSIS in INS-1 cells is improved.

In human hepatic HepG2 cells, schisandrin C isolated from *Schisandra chinensis* was found to increase glucose uptake, but schisandrol A show no activity [11]. Although improving effects of schisandrin C on glucose uptake in hepatic cells, its effect in skeletal muscle cells has not been reported. Here our study showed that schisandrin C increased glucose uptake activity. This effect seemed to be dependent on phosphorylation of IRS-1 and AMPK, which involved PI3K/Akt pathway. We also found that schisandrin C increased expression of GLUT-4. In skeletal muscle, there are two main mechanisms enhancing glucose absorption by haptic tissue. Insulin binding to the insulin receptor leads to phosphorylation of IRS-1. It accelerates phosphorylation of PI3K/Akt leading to activation of GLUT-4 [32,33]. Also, AMPK as upstream of Akt has been reported to activation of GLUT-4 [34]. However, some other study reports that AMPK leads to activation of GLUT-4 independent of Akt [35]. GLUT-4 as a glucose carrier leads to increase in glucose uptake by skeletal muscle to control blood glucose [36]. Therefore, when the expression of these proteins is enhanced after treatment with schisandrin C, glucose uptake in C2C12 cells is improved. However, since schisandrin C exists as a minor constituent in plants, it remains difficult to isolate sufficient quantities for animal experiments, and cell-based assays for investigating the anti-diabetic mechanism of schisandrin C are necessary.

## 4. Materials and Methods

### 4.1. Cell Culture and Chemicals

INS-1 cells (Biohermes, Shanghai, China), a rodent insulin-secreting β-cell line, were cultured in Roswell Park Memorial Institute 1640 medium (Cellgro, Manassas, VA, USA) containing 0.05 mM 2-mercaptoethanol, 2 mM L-glutamine, 1 mM sodium pyruvate, 11 mM D-glucose, 1% penicillin/streptomycin (P/S), 10 mM HEPES, and 10% fetal bovine serum (FBS) at 5% CO_2_ at 37 °C. C2C12 skeletal muscle cells (American Type Culture Collection, Manassas, VA, USA) were cultured in Dulbecco’s modified Eagle’s medium (DMEM, Cellgro) containing 1% P/S and 10% FBS at 5% CO_2_ at 37 °C. The samples used in the present study, schisandrol A, schisandrol B, and schisandrin C, were isolated and purified previously. The purity of these compounds was determined to be over 98% by UHPLC-UV chromatography (see Supplementary Materials).

### 4.2. Cell Viability Assay

INS-1 cells were plated in 96-well plates and cultured overnight, then treated with schisandrol A, schisandrol B, or schisandrin C for 24 h. C2C12 cells were plated in 96-well plates and cultured overnight, then treated with schisandrin C for 24 h. Subsequently, cell viability was determined using the Ez-Cytox cell viability detection kit (Daeil Lab Service Co., Seoul, Korea). Briefly, the cells were incubated in 10 μL Ez-Cytox reagent for 2 h [37]. After incubation, absorbance values at 490 nm were measured using a PowerWave XS microplate reader (Bio-Tek Instruments, Winooski, VT, USA).

### 4.3. Glucose-Stimulated Insulin Secretion (GSIS) Assay

INS-1 cells were plated in 12-well plates and cultured overnight, then washed twice with Krebs-Ringer bicarbonate HEPES buffer (KRBB; 4.8 mM KCl, 129 mM NaCl, 1.2 mM KH_2_PO_4_, 1.2 mM MgSO_4_, 2.5 mM CaCl_2_, 10 mM HEPES, 5 mM NaHCO_3_, and 0.1% BSA, pH 7.4). The culture medium was then replaced with fresh KRBB. After 2 h, the cells were treated with schisandrol A, schisandrol B, schisandrin C, gliclazide (positive control), nifedipine (L-type Ca^2+^ channel blocker), Bay K 8644 (L-type Ca^2+^ channel activator), diazoxide (K^+^ channel activator), or glibenclamide (K^+^ channel blocker) for 2 h. Subsequently, the cells were incubated in basal (2.8 mM) and stimulant (16.7 mM) concentrations of glucose for 1 h, and the glucose-stimulated insulin secretion (GSIS) was assessed with a rat insulin ELISA kit. GSIS was expressed as the glucose stimulation index (GSI), which was calculated by dividing the insulin concentration at 16.7 mM glucose by the insulin concentration at 2.8 mM glucose.

### 4.4. Western Blot Analysis

INS-1 cells and C2C12 cells were plated in 6-well plates and cultured overnight, then treated with schisandrin C for 24 h. Subsequently, the cells were lysed with RIPA buffer (Cell Signaling, Danvers, MA, USA) for 20 min. Equal amounts of protein were separated by size using 10% sodium dodecyl sulfate-polyacrylamide gel [38]. Separated proteins were transferred by electroblotting to polyvinylidene difluoride (PVDF) membranes. The PVDF membranes were probed with the primary antibodies (Cell Signaling) overnight at 4 °C, then incubated with horseradish peroxidase (HRP)-conjugated anti-rabbit secondary antibodies (Cell Signaling) for 1 h at 4 °C, and with enhanced chemiluminescence reagent (GE Healthcare UK Limited, Buckinghamshire, UK) for 5 min at room temperature. Proteins were detected using a chemiluminescence system (FUSION Solo, PEQLAB Biotechnologie GmbH, Erlangen, Germany).

### 4.5. ADP/ATP Ratio Assay

INS-1 cells were plated in 12-well plates and cultured overnight, then washed twice with Krebs-Ringer bicarbonate HEPES buffer (KRBB; 4.8 mM KCl, 129 mM NaCl, 1.2 mM KH_2_PO_4_, 1.2 mM MgSO_4_, 2.5 mM CaCl_2_, 10 mM HEPES, 5 mM NaHCO_3_, and 0.1% BSA, pH 7.4). The culture medium was then replaced with fresh KRBB. After 2 h, the cells were treated with schisandrin C for 2 h. Subsequently, the cells were incubated in basal (2.8 mM) and stimulant (16.7 mM) concentrations of glucose for 1 h, and ADP/ATP ratio in cell lysates was determined using ADP/ATP ratio assay kit (Sigma Aldrich, St. Louis, MO, USA) according to the manufacturer’s instructions.

### 4.6. Glucose Uptake Assay

Differentiation of C2C12 cells into myotubes was performed in DMEM containing 1% P/S and 2% horse serum as previously described [39]. After 4 days, treatment was performed in DMEM containing 1% P/S, 2% horse serum, 10% FBS, and 2% bovine serum albumin in the absence or presence of schisandrin C for 16 h. Glucose uptake activity was examined by 2-(N-(7-nitrobenz-2-oxa-1,3-diazol-4-yl) amino)-2-deoxyglucose (2-NBDG) uptake assay kit (Sigma-Aldrich) as described in the manufacture’s protocol.

### 4.7. Statistical Analysis

Statistical significance was determined using one-way analysis of variance (ANOVA) and multiple comparisons with Bonferroni correction. Statistical significance was set at *p* < 0.05. All analyses were performed using SPSS Statistics ver. 19.0 (SPSS Inc., Chicago, IL, USA).

## 5. Conclusions

The present study demonstrated that schisandrin C can induce GSIS in INS-1 cells in vitro. INS-1 cells treated with schisandrin C showed increased protein expression of PPARγ, PDX-1 and phosphorylation levels of IRS-2, PI3K, Akt, which was demonstrated to have important roles in these effects. In addition, schisandrin C can improve glucose uptake in C2C12 cells in vitro. C2C12 cells treated with schisandrin C showed increased protein expression of GLUT-4 and phosphorylation levels of IRS-1, AMPK, PI3K, Akt, which was demonstrated to have important roles in these effects. More studies and animal experiments are needed to fully investigate other signaling pathways and its mechanism of action. These results may provide evidence that schisandrin C treatment may be beneficial in devising novel anti-T2D strategies.

## Supplementary Materials

Figure S1: Effect of schisandrin C on the protein expression levels of peroxisome proliferator-activated receptor γ(PPARγ), pancreatic and duodenal homeobox 1 (PDX-1), phospho-insulin receptor substrate-2 (P-IRS-2) (Ser731), IRS-2, phospho-phosphatidylinositol 3-kinase (P-PI3K), PI3K, phospho-Akt (P-Akt) (Ser473), and Akt, Figure S2: Effects of schisandrin C on the glucose uptake in C2C12 cells. (C) Effect of schisandrin C on the protein expression levels of phospho-insulin receptor substrate-1 (P-IRS-1), IRS-1, phospho-AMP-activated protein kinase (P-AMPK), AMPK, phospho-phosphatidylinositol 3-kinase (P-PI3K), PI3K, phospho-Akt (P-Akt) (Ser473), Akt, glucose transporter type 4 (GLUT-4), and glyceraldehyde 3-phosphate dehydrogenase (GAPDH) in INS-1 cells treated or untreated with 2.5 and 5 μM schisandrin C for 24 h.
