# Supplementary material for: Schisandrin C Affects Glucose-Stimulated Insulin Secretion in Pancreatic β-Cells and Glucose Uptake in Skeletal Muscle Cells
Source: Molecules. 2021 Oct 28;26(21):6509. doi: 10.3390/molecules26216509 (PMC8587027; doi:10.3390/molecules26216509)
Supplement: Supplementary file 1 [file molecules-26-06509-s001.zip › molecules-1440658-supplementary.pdf]

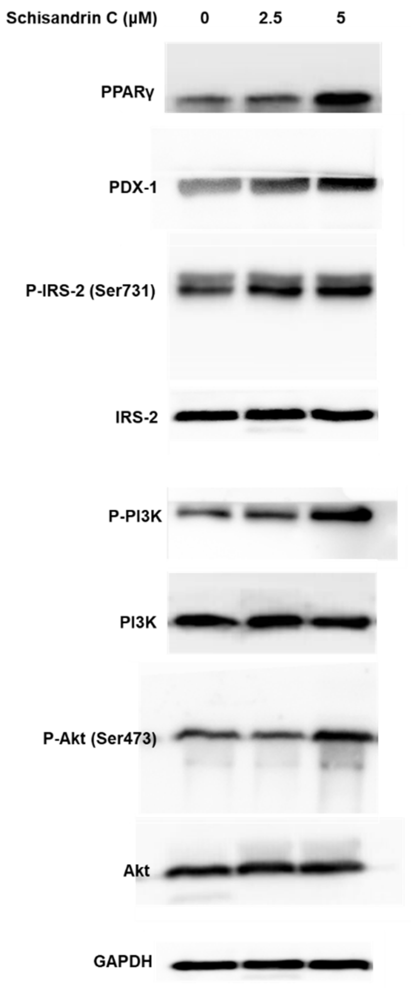

Figure S1. Effect of schisandrin C on the protein expression levels of peroxisome proliferator-activated receptor γ (PPARγ), pancreatic and duodenal homeobox 1 (PDX-1), phospho-insulin receptor substrate-2 (P-IRS-2) (Ser731), IRS-2, phospho-phosphatidylinositol 3-kinase (P-PI3K), PI3K, phospho-Akt (P-Akt) (Ser473), and Akt.

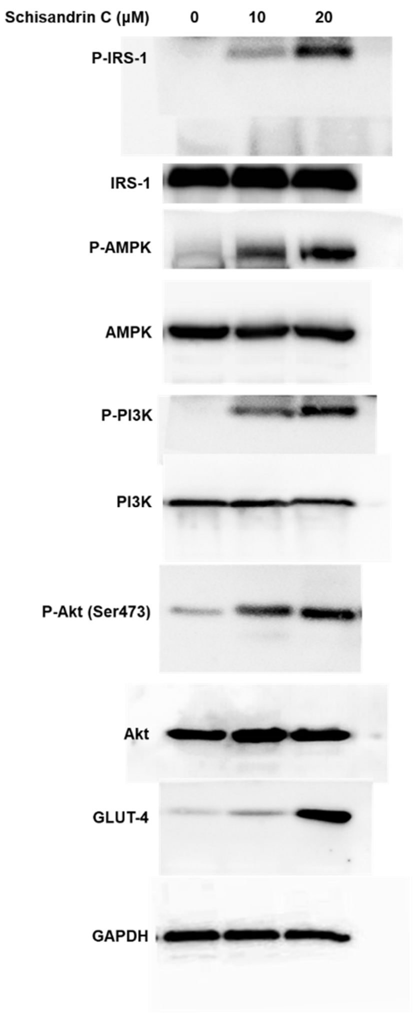

Figure S2. Effects of schisandrin C on the glucose uptake in C2C12 cells. (C) Effect of schisandrin C on the protein expression levels of phospho-insulin receptor substrate-1 (P-IRS-1), IRS-1, phospho-AMP-activated protein kinase (P-AMPK), AMPK, phospho-phosphatidylinositol 3-kinase (P-PI3K), PI3K, phospho-Akt (P-Akt) (Ser473), Akt, glucose transporter type 4 (GLUT-4), and glyceraldehyde 3-phosphate dehydrogenase (GAPDH) in INS-1 cells treated or untreated with 2.5 and 5 μM schisandrin C for 24 h.
